# Assessing Electronic Health Record (EHR) Use during a Major EHR Transition: An Innovative Mixed Methods Approach

**DOI:** 10.1007/s11606-023-08318-w

**Published:** 2023-10-05

**Authors:** Brianne Molloy-Paolillo, David Mohr, Deborah R. Levy, Sarah L. Cutrona, Ekaterina Anderson, Justin Rucci, Christian Helfrich, George Sayre, Seppo T. Rinne

**Affiliations:** 1 Center for Healthcare Organization and Implementation Research (CHOIR), VA Bedford Healthcare System, Bedford, MA USA; 2 Center for Healthcare Organization and Implementation Research (CHOIR), VA Boston Healthcare System, Boston, MA USA; 3 Boston University School of Public Health, Boston, MA USA; 4 Center of Innovation for Pain Research, Informatics, Multimorbidities, and Education (PRIME), VA Connecticut Health Care, West Haven, CT USA; 5 Yale University School of Medicine, New Haven, CT USA; 6 Department of Population and Quantitative Health Sciences/Division of Health Informatics and Implementation Science, UMass Chan Medical School, Worcester, MA USA; 7 Division of Pulmonary Critical Care, Boston University, Boston, MA USA; 8 Seattle-Denver Center of Innovation, VA Puget Sound Health Care System, Seattle, WA USA; 9 Health Systems and Population Health, School of Public Health, University of Washington, Seattle, WA USA; 10 Pulmonary & Critical Care Medicine, School of Medicine, Boston University, Boston, MA USA

**Keywords:** electronic health records, EHR transition, EHR use metrics, usability, mixed methods

## Abstract

**Background:**

Electronic health record (EHR) transitions are inherently disruptive to healthcare workers who must rapidly learn a new EHR and adapt to altered clinical workflows. Healthcare workers’ perceptions of EHR usability and their EHR use patterns following transitions are poorly understood. The Department of Veterans Affairs (VA) is currently replacing its homegrown EHR with a commercial Cerner EHR, presenting a unique opportunity to examine EHR use trends and usability perceptions.

**Objective:**

To assess EHR usability and uptake up to 1-year post-transition at the first VA EHR transition site using a novel longitudinal, mixed methods approach.

**Design:**

A concurrent mixed methods strategy using EHR use metrics and qualitative interview data.

**Participants:**

141 clinicians with data from select EHR use metrics in Cerner Lights On Network®. Interviews with 25 healthcare workers in various clinical and administrative roles.

**Approach:**

We assessed changes in total EHR time, documentation time, and order time per patient post-transition. Interview transcripts (n = 90) were coded and analyzed for content specific to EHR usability.

**Key Results:**

Total EHR time, documentation time, and order time all decreased precipitously within the first four months after go-live and demonstrated gradual improvements over 12 months. Interview participants expressed ongoing concerns with the EHR’s usability and functionality up to a year after go-live such as tasks taking longer than the old system and inefficiencies related to inadequate training and inherent features of the new system. These sentiments did not seem to reflect the observed improvements in EHR use metrics.

**Conclusions:**

The integration of quantitative and qualitative data yielded a complex picture of EHR usability. Participants described persistent challenges with EHR usability 1 year after go-live contrasting with observed improvements in EHR use metrics. Combining findings across methods can provide a clearer, contextualized understanding of EHR adoption and use patterns during EHR transitions.

**Supplementary Information:**

The online version contains supplementary material available at 10.1007/s11606-023-08318-w.

## Introduction

Transitions between electronic health record (EHR) systems are becoming more prevalent due to technological advances, hospital consolidations, and government incentives.^1,2^ EHR transitions are complicated and resource-intensive for organizations. They can also be particularly challenging for healthcare workers who must unlearn their prior EHR workflows, adapt quickly to the new system, and continue to provide high-quality patient care. Despite their complexity, EHR transitions are understudied. Measurement of healthcare personnel’s actual use of new EHRs as well as their perceptions of EHR usability are important areas that could inform improvement efforts, yet are poorly understood.^1^

EHR log data is a rich source of information that can be used to evaluate healthcare workers’ EHR use during EHR transitions. This data captures and timestamps user activity within the EHR,^3–5^ and is generated as a byproduct of routine patient care, requiring no additional engagement from healthcare workers. EHR log data is processed by vendor EHR products into EHR use metrics, such as those available in Cerner Lights On Network®.^3,6^ Common EHR time-based use metrics include total time in the EHR, time on note documentation, time on inbox, time on prescriptions, and time spent on work outside of work hours.^7^ Given the availability of EHR use metrics, health systems may be interested in using this data to assess EHR transition progress and healthcare workers’ proficiency with the new system.^8^

Health systems seeking to use EHR metrics should keep in mind that the advantage of this passive form of data collection is also an important limitation – without healthcare workers’ input, EHR use metrics lack vital contextual information about the users’ experience in general and especially during EHR transitions when users are learning a new EHR.^4,8^ Complementing EHR use metrics with a qualitative assessment of frontline workers’ perspectives provides a more complete understanding of the end user experience during EHR transitions.^9^

The Department of Veterans Affairs (VA), the largest nationally integrated healthcare system in the U.S., is in a unique position to improve our understanding of user experience with EHR transitions. It is currently undergoing a nationwide, 10-year EHR transition replacing its homegrown EHR with a vendor-based EHR from Oracle Cerner (“Cerner”), an endeavor that represents one of the biggest EHR transitions in history.^10^ VA’s organization-wide EHR-to-EHR transition offers a valuable opportunity to examine EHR usability while exploring and validating EHR use metrics.

## Objectives

The key objectives of this study were to: 1) assess EHR usability and uptake at VA’s first EHR transition site, and 2) develop a novel longitudinal, mixed methods approach to studying EHR use during EHR transitions that integrates EHR use metrics and qualitative interview data. By applying this mixed methods approach, we sought to provide a rich description of end user experience with the new EHR. We hypothesized there would be a gradual decline in the time needed to perform EHR tasks as clinicians become more skilled in the new EHR. We also expected that interview data with healthcare workers would reflect increasingly positive perceptions of the EHR system over time in line with improvements in EHR use metrics.

## Methods

This study represents one component of a larger, multi-year evaluation project of the EHR modernization effort at VA.^11^ This evaluation was designated as non-research/quality improvement by the VA Bedford Healthcare System Institutional Review Board.

We conducted a mixed methods analysis of EHR usability with quantitative EHR use metrics and qualitative interview data from the Mann-Grandstaff VA Medical Center in Spokane, WA, the first VA site to implement Cerner. Our analysis covers November 2020 to November 2021, representing the 12-month period following the site’s go-live date (10/24/20). This data collection timeline was developed in collaboration with strategic partners.

## Participants

### Quantitative

We included 141 physicians (MDs and DOs) with post-transition EHR use metrics available in Cerner Lights On Network®, a data analytic platform with automatically generated data based on user interactions in the EHR.^6^ There were 127 MDs and 14 DOs of which 111 practiced at the main medical center and 30 worked at a VA community based outpatient clinic. Physicians were from the following specialty areas: Emergency (n = 18), Inpatient (n = 23), Medical (n = 14), Primary Care (n = 39), Mental Health (n = 17), Surgical (n = 17), and Multiple Areas (n = 13).

### Qualitative

We focused on a subset of 90 post-EHR transition interviews and brief check-ins with 25 clinicians and staff in various roles (e.g., physicians, pharmacists, nurses, medical support assistants, and allied health workers) (Table 1).

**Table 1:** Post-EHR Transition Qualitative Data Collection

Interviewees (n = 25)	Check-ins	2 Months Post-Go-Live Interviews	10 Months Post-Go-Live Interviews	Total Interviews
Leadership and clinicians^a^	22	14	13	49
Nurses^b^	12	5	5	22
Staff^c^	13	4	2	19
Total	47	23	20	90

^a^Physicians, Clinical Pharmacists, Psychologists

^b^RNs and LPNs

^c^Medical assistants, phlebotomists, counselors, audiologists, physical therapists

*Interviews were virtually conducted using MS Teams. 2-month and 10-month interviews were approximately 60 minutes long and brief check-ins lasted about 15 to 30 minutes*

## Data Collection Approach

### Quantitative

The Cerner Lights On Network® contains user log data on time and duration of EHR tasks post-transition and transforms them into EHR use metrics (an example of vendor-derived metrics). We accessed Lights On data for three metrics that reflect physicians’ use of the new EHR: 1) Total time in EHR per patient seen (i.e., the amount of active time spent reviewing a patient’s chart in minutes), 2) time on orders (average time across all basic order workflows calculated per patient in minutes including time placing orders through favorites, folders, and search) and 3) documentation time per patient seen in minutes (e.g., the total time to complete documentation for a patient). We selected these metrics because they are proposed components of EHR use measurements in the literature, are readily available in the Lights On Network, and represent key metrics of interest for VA leadership.^7,12,13^ We chose total EHR time to illustrate a global assessment of EHR use. We focused on order and documentation time as both were frequently described in qualitative interviews. Direct pre-implementation comparison metrics were not available from the legacy EHR system.

### Qualitative

Between July 2020 and November 2021, a total of 90 semi-structured interviews (~ 60 min) and brief “check-ins” (~ 15–30 min) with 25 healthcare workers were conducted, immediately after the transition (1–3 months after) and again 10–12 months after the transition (Table 1). Check-ins were intentionally short to limit participant burden. Not all participants provided data at each data collection point (i.e., 80% of post-go-live interviewees completed at least one check-in, 68% completed more than one check-in, 92% completed the initial post-go-live interview, and 80% participated in the 10–12 month interview). We used snowball sampling for recruitment by asking local leaders to refer groups of individuals from clinical teams. Prospective participants were emailed an invitation requesting their voluntary participation in interviews about their EHR transition experiences at multiple timepoints. Once enrolled, participants were asked to provide further contacts who might be willing to participate. Interviews and check-ins were conducted virtually on MS Teams® by experienced qualitative researchers. Interviews were audio-recorded with verbal consent from the participant and professionally transcribed.

Semi-structured interview guides with grounded probes were used for data collection (Appendix 1). Grounded probes were standard prompts with a stemmed format (e.g., What do you mean by ____?) that were completed using participants’ own language to elicit additional information if a participant’s initial response was limited or required clarification.^14,15^ Interview guides were iteratively designed based on team discussion involving skilled qualitative researchers. The goal of the interviews was to elicit information about participants’ personal experiences with the EHR transition and the new Cerner EHR, including their perceptions of EHR usability.

## Data Analysis

### Quantitative

EHR use metrics were extracted from the Cerner Lights On Network and analyzed using SAS statistical software. We ran descriptive statistics (means and standard deviations) to summarize measures and examined longitudinal changes in EHR metrics over 12-months post-EHR transition. We did not examine differences in EHR use metrics by physician specialty area or facility setting for this analysis.

### Qualitative

A combination of deductive and inductive content analysis approaches was used for analysis. ^16^ We generated a list of a priori categories (e.g., EHR support, EHR training, software functionality, impact on Veterans) reflecting the project aims and conducted line-by-line coding of all transcripts in ATLAS.ti 9. To calibrate each analysts’ approach to coding and ensure coding rigor,^17^ our team selected one transcript for coding by all analysts in ATLAS.ti. Following coding of this transcript, we reviewed, discussed, and resolved discrepancies as a group. Each subsequent transcript was coded by one analyst and new analysts’ coded transcripts were reviewed by the lead, senior methodologist until coding approaches were appropriately aligned. The team developed consensus around code categories and reconciled revised codes and categories in weekly meetings. New codes and code groups were added throughout the coding process to reflect emergent concepts gleaned from the data. Analysts were encouraged to write analytic memos and use code comments to define emergent codes for group discussion. The qualitative analysis team met weekly to discuss impressions from the data, review analytic memos, and resolve challenges related to coding. After the team identified EHR use and usability as a topic of interest for focused analysis, the first author reviewed all coded passages relevant to EHR usability and generated initial themes that were subsequently developed and refined with the co-authors' input in a process of content analysis.

### Mixed Methods

We employed a concurrent mixed methods strategy with mixing occurring during interpretation.^18,19^ Quantitative and qualitative data were collected in parallel and analyzed separately. Both quantitative and qualitative results were then compared to draw conclusions at the interpretation stage in the discussion. A greater emphasis was placed on qualitative findings due to the depth of available interview data capturing the lived experiences of frontline healthcare workers and in order to validate and assess convergence with patterns observed in the EHR use metrics.^19^

## Results

### Quantitative

**Figure 1:**
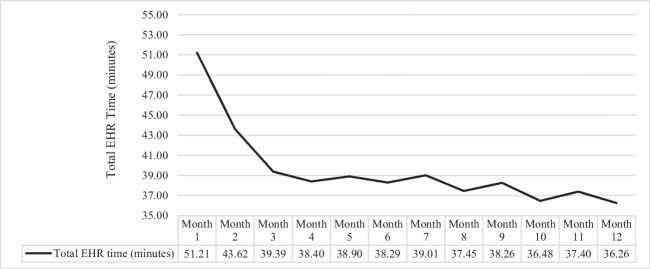
**Mean Total EHR Time per Patient (minutes) by post-EHR transition month.**

**Figure 2:**
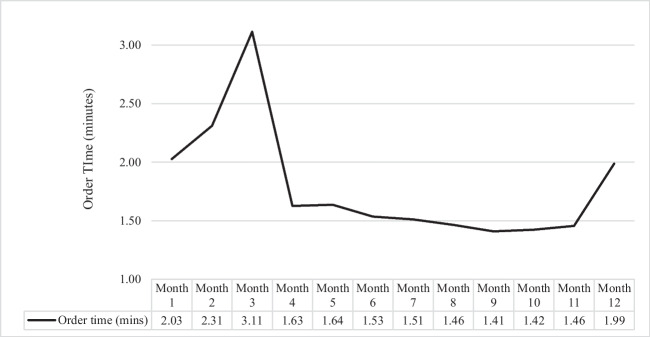
**Mean Order Time per Patient (minutes) by post-EHR transition month.**

**Figure 3:**
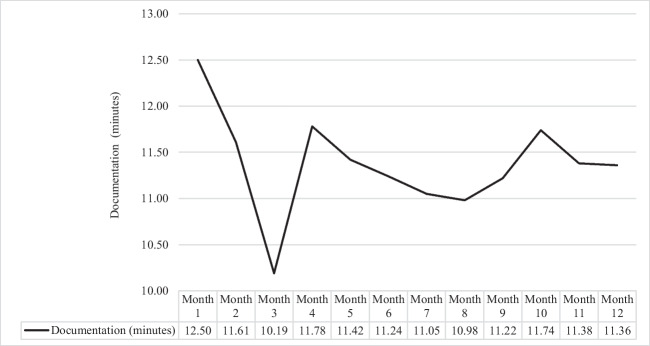
**Average Documentation Time per Patient Seen (minutes) by post-EHR transition month.**

The mean total time spent in the EHR post-transition was 39.56 min (*SD* = 4.12), the mean order time was 1.79 min (*SD* = 0.50), and average time spent documenting for a patient was 11.37 min (*SD* = 0.55). We observed a rapid decline in Total EHR time from month 1 (*M* = 51.21) to 4 (*M* = 38.40), followed by a pattern of stabilization (see Fig. 1). Order time displayed a sharp increase from month 1 (*M* = 2.03) to month 3 (*M* = 3.11), followed by a steep decrease at month 4 (*M* = 1.63) (see Fig. 2). Documentation time also exhibited a drastic decline from month 1 (*M* = 12.50) to 3 (*M* = 10.19) (see Fig. 3). Overall, physicians spent less time in the EHR per patient (*M*_*month12*_ = 36.26 vs. *M*_*month1*_ = 51.21), on documentation (*M*_*month12*_ = 11.36 vs. *M*_*month1*_ = 12.50), and entering orders (*M*_*month12*_ = 1.99 vs. *M*_*month1*_ = 2.03) at 12-months compared to 1-month post-transition.

### Qualitative

Most interview participants expressed concerns with the new EHR’s usability and functionality throughout the first year following the EHR transition. We identified four themes in participants’ accounts: 1) Clinical tasks took longer than in the previous system and created frustration, 2) Inefficiencies stemmed from both insufficient training and inherent features with the system, 3) Growing mastery and use of time-saving functions contributed to modest usability improvements, and 4) Clinicians had difficulties with EHR usability throughout the transition, and these challenges persisted up to a year after go-live. Exemplar quotes are presented with each theme along with the participant’s unique ID number, professional role, and the timepoint of the interview.

#### Theme 1: Clinical Tasks Took Longer Than in the Previous System and Created Frustration

Many participants reported existing tasks (e.g., orders, referrals) taking longer in Cerner, compared to the legacy system, CPRS.

“...it’s not causing a stop in care, but something that could have taken 15 seconds is now taking 3 minutes.” (101, Nurse, One-Month Post-Go-Live)

Several participants also noted that the new system required extra clicks, which extended the time needed to complete tasks.

“It still does take longer… if it took 5 minutes in CPRS, in Cerner, when we first started, it took 45 minutes. And now that 5 minutes takes 25 minutes… At this point now it’s just the system; we’re familiar with what we have to do, it’s just all of the extra clicks.” (101, Nurse, Two-Months Post-Go-Live)

“It’s a lot of work on our end, ... clicking and pointing and opening windows. It’s just a longer process.” (102, Clinical Staff, Two-Months Post-Go-Live)

“But there are a lot of things that just take a lot of extra clicks. Like placing a referral, there’s like double the work to do that. That’s ...one of the big ones that’s kind of time consuming.” (103, Clinician, Two-Months Post-Go-Live)

Some expressed frustration that everyday tasks took longer even a year later:

“It is not an advancement from the CPRS charting system that we had. It is causing us a lot more time to do pretty much everything in our normal everyday jobs. And adding to pretty much everyone’s frustration across the board at the VA.” (104, Clinician, 10-Months Post-Go-Live)

#### Theme 2: Inefficiencies Stemmed from both Insufficient Training and Inherent Features with the System

Participants cited multiple reasons for inefficiencies from inadequate training to inherent features of the software. Many participants described the new EHR as not intuitive and requiring more steps or “mouse clicks” (106, Clinician, One Month Post-Go-Live). The phenomenon of having multiple ways to do the same thing, some of them more efficient than others, was also noted. Participants also described the new EHR as messy and cluttered, which made it hard to search for things like medications.

“…it’s a very complex system, a lot of clicking. It’s not intuitive, you just have to use it over and over and over before you finally remember it. …some of the training helped <but> we did not have Referral Management training before go live, we did not have med reconciliation training before go live. So, those have been challenging areas where we’ve had to figure it out as we go, and try to do some training after go live, which is not optimal.” (107, Clinician, Two-Months Post-Go-Live)

“…a lot of times it’s difficult to find things. Like yesterday I was just trying to find Vitamin B Complex with Vitamin C, and I just couldn’t find it. In Cerner there’s about 400 options, and none of them are what I needed. So it’s just digging through a lot to find things, it just takes a long time.” (105, Clinician, Two-Months Post-Go-Live)

One participant summarized, searching for notes was “like finding a needle in haystack” (105, Clinician, 10-Months Post-Go-Live).

Participants reported that the system’s efficiency did not match what they had been promised and described this challenge as independent of the user’s level of system familiarity.

“I know during training we were all excited because they said it’d be a lot fewer clicks, that was the advertisement, you can get the same thing done in fewer clicks. And what we’re finding is that’s actually not true, you get the same thing done in like 5 times as many clicks. So it’s something that’s not even about the familiarity with the program, it’s just how the program is built.” (101, Nurse, One Month Post-Go-Live)

Finally, some participants commented on inefficiencies stemming from new task processes. For example, the new process for documenting workload between actual visits was seen as confusing and labor-intensive:

“But, just to renew one medication on a patient that you’re not seeing that day, you have to create what’s called an in-between visit, which is like 10 steps to create that in-between visit so that you can renew their medicine, and then another 10 steps to renew the medicine. So it’s just extremely, you know, taking a lot of time.” (105, Clinician, One Week Post-Go-Live)

#### Theme 3: Growing Mastery and Use of Time-Saving Functions Contributed to Modest Usability Improvements

Some participants noted modest improvements, which were attributed to growing experience and mastery, as well as to time-saving shortcuts in the system like auto-populated text (i.e., dot phrases), a quicker signing process, and setting up favorites.

“…there’s some really good things, like when I sign my name, I just click the word sign. Before I had to put in my signature numbers, right? So it’s probably saving me 1,000 clicks a day. It’s slick, once you get used to it.” (108, Clinician, Two-Months Post-Go-Live)

“…I can actually chart pretty quickly. It’s all of the broken links, the stuff that doesn’t work, that causes the problem. And then they go in and fix something, and it’s a problem. It is internet based.” (110, Clinical Staff, 10-Months Post-Go-Live)

“Like dot phrases. So instead of, if I want to put in my preferred physical exam, or review of systems, or my kind of preop or whatever, I can do like a backslash preop, and it’ll just populate in, so I don’t have to type it all out. So, I think they call them dot phrases within Cerner, there’s a lot of them that are in Cerner that anyone can use...you can actually make your own as well.” (105, Clinician, Two-Months Post-Go-Live)

One participant expressed optimism that EHR tasks like placing a consult would become easier with practice.

“[In CPRS]… It took like 5 minutes, 10 minutes. 5 minutes. This one took me half an hour to figure out, I anticipate that will get easier as I have to do more of them.” (106, Clinician, Three Weeks Post-Go-Live)

#### Theme 4: Clinicians had Difficulties with EHR Usability Throughout the Transition and These Challenges Persisted up to a Year After Go-Live

Approximately one-year post-transition, many participants reported continual issues with functionality. Numerous participants commented on the system malfunctioning, noting that some features appeared to be broken, nonexistent, or inconsistent throughout the transition.

“I am still struggling with this program. And there are things that still aren’t built. We can’t receive consults, so we’ve had to devise some workarounds. There’s a lot of things that I still don’t know how to do that are basic functions of my job that aren’t built yet and not working well.” (106, Clinician, Three Weeks Post-Go-Live)

Several others described the new EHR as an unstable and unreliable system and noted regularly occurring system glitches interfering with their ability to do their job.

“…this is just not a stable system. Like I said, 2 ½ hours this morning of complete and total frozen system. And… we’re getting errors all day, every day. … So then we’ve got to pause our work, identify…who puts in a ticket…It’s constant. … I mean, it’s pretty rare to have just a normal flow day where you just do your work and not worry about the reliability of the system you’re doing it on.” (111, Clinician, 10-Months Post-Go-Live)

“…it seems like over the last week we’ve had a rash, probably 10 days now, a lot of people having difficulty maybe signing on, or it being really glitchy, or freezing, or having to restart. And it seems to be global.”(112, Clinician, 10-Months Post-Go-Live)

## Discussion

The present study used EHR use metrics and qualitative data to conduct a novel mixed methods assessment of EHR usability and user experience at VA’s first EHR transition site. To understand post-transition EHR use, we examined three EHR time-based metrics and contextualized them with data from healthcare worker interviews. As we hypothesized, EHR use metrics demonstrated gradual improvements over the 12 months (e.g., decreased time spent in the EHR and on documentation in addition to a slight decrease in time spent entering orders). However, longitudinal EHR metrics still painted an incomplete picture of EHR usability and uptake. In our interviews, healthcare workers consistently reported challenges with EHR usability and functionality, which persisted roughly one-year post-transition. Our mixed methods approach yielded a more nuanced interpretation of findings than we would have achieved with either methodology alone, lending insight into EHR usability conditions that differed from our expectations.

While EHR use metrics have been used to assess clinician performance^7^ and determine the impact of improvement initiatives,^9,20^ our work indicates that these metrics may not always reflect EHR end users’ experiences. Qualitative data can add critical information to contextualize EHR use metrics to better understand healthcare worker experiences, especially during EHR transitions when there are rapid changes in EHR metric values. In our study, qualitative data clarified and deepened our understanding of healthcare workers’ frustrations with the new EHR despite improvements in EHR data. Combining both methods can generate insights that can inform targeted training and EHR system redesign efforts, allowing health systems to improve clinician experience and enhance care delivery efficiency.^9^

EHR transitions are often justified as an effort to improve usability and efficiency from aging systems, but our study along with past studies indicate that they do not always live up to these promises. For example, our study supports findings from two longitudinal studies which demonstrated that most physicians’ opinions about a new EHR worsened following the transition and failed to return to baseline levels up to two years later.^21,22^ Specifically, healthcare professionals’ perceptions of the new EHR remained largely negative up to a year later and did not correspond with observed improvements in EHR use metrics. An assessment period greater than two years would be valuable to investigate whether the alignment increases in the longer term and may reveal additional improvements. However, there is evidence to suggest that documentation may be less impacted than other EHR tasks. Hanauer et al. (2017) found that documenting patient visits was the only measure remaining above baseline, which is consistent with the present study’s qualitative findings indicating that documentation in the new EHR was just as efficient or more efficient than the old EHR.^17^ The EHR use metric for documentation time also showed notable improvements over time. Yet, taken as a whole, participants reported that EHR use issues and inefficiencies were vast and went beyond the point of being a potential user problem signaling concern about the EHR system’s build and functionality.

### Limitations

This study has several limitations. Time-based EHR use metrics were available only after the Cerner EHR transition; therefore, direct pre-implementation comparisons were not possible. The lack of comparable and standardized EHR use metrics across EHRs and vendors is a known challenge in the field presenting difficulties in making pre/post comparisons during transitions.^12^ Because we did not collect EHR use metrics or comparative qualitative data for the CPRS EHR being replaced, we cannot offer a detailed comparison between the new EHR and the legacy EHR. However, many interviewees reported existing tasks (e.g., orders, referrals) taking longer in Cerner, compared to the previous system. EHR use metrics were accessed for physicians at the site, while the qualitative data was from a larger group of healthcare workers. Future work could examine EHR use metrics for non-physicians (e.g., other independent licensed practitioners and nurses). While EHR use data is a powerful tool for understanding early patterns of EHR uptake following an EHR transition, vendor-derived EHR use metrics, such as those on Cerner Lights On Network®, may oversimplify complex EHR interactions^5,8^ and restrict detailed examination of the data produced during EHR use. Examining how vendor metrics compare to raw log and interview data could provide further insight into why our quantitative and qualitative findings did not align. Future work is also needed to examine the application of vendor-derived metrics in diverse settings as these metrics have mainly been designed to measure EHR use in ambulatory care settings, and they have not been validated across the spectrum of clinical care.^9^ Additionally, patterns of EHR use for one transition site may not resemble patterns for others, due to factors related to unique site characteristics, the version of the product, or the general state of the transition itself. Snowball sampling for qualitative interviews is subject to selection bias; we made efforts to mitigate selection bias by recruiting participants from diverse roles. Lastly, due to time constraints, we did not revisit and recode previously analyzed transcripts with emergent codes, which may have resulted in a less comprehensive categorization of the data.

### Future Directions for Research

This novel mixed-methods approach offers critical lessons that could inform future user experience evaluations, including: (1) EHR use data must be validated with alternate methods; (2) combining EHR use metrics and interview narratives enhances understanding of end user experience; (3) qualitative data helps identify usability issues and contextual factors; and (4) mixed methods can inform targeted interventions for quality improvement. In our study, EHR use metrics related to order time and documentation time both demonstrated month-to-month variability that may relate to random variation, system updates, and policy changes rather than familiarity and usability of the new EHR. This variance underscores the need for future research to look at longer longitudinal trends and complement these data with other methods. Furthermore, future mixed methods studies could enhance live monitoring of EHR use metrics by using targeted qualitative interviews aimed at understanding and addressing unusual trends in EHR use metrics. We did not employ pre-existing technology frameworks to this work, but future studies building on our methodology might benefit from applying the Technology Acceptance Model or the Unified Theory of Acceptance and Use of Technology (to further inform understanding of perceived ease of use and acceptance) and should also consider additional influential factors affecting healthcare technology uptake (e.g. anxiety, computer self-efficacy, innovativeness and trust).^23^ Future research may also consider using unique identifiers to link EHR use metrics with qualitative interview or survey data to examine trends in EHR use metrics by physician specialty, amount or type of EHR training, and prior exposure to the Cerner EHR.

## Conclusions

Applying an innovative mixed methods approach that integrated EHR use metrics and qualitative data provided a rich, nuanced picture of EHR usability for healthcare workers at VA’s first transition site. Longitudinal assessments of EHR use metrics and user experience during an EHR transition can yield vital information on the course of EHR transitions. Our findings suggest that policy makers should not rely on vendors' EHR use metrics alone to evaluate the status of an EHR transition as they do not offer a full picture of EHR usability as evidenced by our mixed methods approach. Healthcare organizations would benefit from eliciting user input about the EHR transition and helping to communicate realistic expectations about usability at future sites. Continuing to use mixed methods to study EHR transitions may inform additional improvements that optimally support workflows and expectation-management at future VA rollout sites and beyond.

### Supplementary Information

ESM 1 (PDF 839 kb)

## Data Availability

Datasets generated and analyzed during this study are not publicly available because they contain information that could compromise participant privacy. Upon reasonable request, the authors may be able to provide anonymized data extracts.
