# Supplementary material for: Assessing Electronic Health Record (EHR) Use during a Major EHR Transition: An Innovative Mixed Methods Approach
Source: J Gen Intern Med. 2023 Oct 5;38(Suppl 4):999–1006. doi: 10.1007/s11606-023-08318-w (PMC10593729; doi:10.1007/s11606-023-08318-w)
Supplement: Supplementary file 1 — (PDF 839 kb) [file 11606_2023_8318_MOESM1_ESM.pdf]

Appendix 1. Post-EHR Transition Qualitative Check-In and Interview Guides.

CHECK-INS POST-IMPLEMENTATION

Status update

- Since the last time we talked, what has been happening in your clinic related to the EHR transition?

Adaptations to implementation

- What's the biggest challenge your team is working on?
  - Are you aware of any [other] efforts to address the issue?

Environment / context

- [Time permitting]: Have there been any other changes at your facility that may affect the EHR transition?

2 MONTHS POST-IMPLEMENTATION

Grounded probes/prompts: *If responses are limited or require clarification, probes may be used to elicit more detailed responses. Probes should use words or phrases presented by the participant using one of the following formats:*

What do you mean by ____________?
Tell me more about___________.
Give me an example of ____________.
Tell me about a time when ____________.
Who ____________?
Where ____________?
What, if anything, was helpful about ________________?
What, if anything, was not helpful about ________________?
What, if anything, made ________________ difficult?
What, if anything, made ________________ easier?
What was the impact of ____________?
Tell me about the _______ training session you participated in.

I know we've been checking in regularly over the past few months – now I want to give you some time to look back at the transition as a whole

1. **Tell me about transitioning to Cerner. (Be prepared to jump around the guide as needed following the lead of the respondent)**

2. **Has the Cerner transition impacted vets?**

   If needed:
   What about Veteran care?
   What about Veteran experience?
   How is the Cerner transition affecting the patient portal?

3. **Information/Communication**
    - Tell me about communication regarding Cerner since go-live.

      AS NEEDED:
        - Tell me about any information you have received from
        - local leaders [from chief or supervisor]
        - VA leadership
        - Cerner.
        - VISN and VAMC-level leadership

4. **Training and education**
    - Did the training you received prior to go-live prepare you to use Cerner?
    - Have you received any additional training since the go-live? Please tell me about it.
      a. If they say "no" - probe – Would you want to receive more training in using Cerner? Why or why not?

5. **Resources and capacity**
    - Tell me about clinic capacity and access for vets since go-live

      AS NEEDED:
      a. Tell me about the Clinical Resource Hub [Boise Hub]
      b. Tell me about the call center
      c. Clinic grids
    - Anything else that was done to support the transition?

6. **Using Cerner**
    - Tell me about using Cerner.
    - Which functions/elements of Cerner you typically use on an average day?

      AS NEEDED
        - How easy is Cerner to use for (scheduling, entering orders, or whatever is relevant to their practice)?
        - Tell me about documentation in Cerner.

      AS NEEDED
        - Walk me through entering a note.
        - Have you made any modifications or changes to Cerner to fit your work needs better?

      IF NOT MENTIONED:
        - Note templates (dot phrases)
        - Customized order sets
        - Dashboards
    - Do you document outside of your regular work hours?
        - *Probe if needed*
    - Tell me about communicating with other VA sites through the EHR.
        - Tell me about Cerner communicating with non-VA systems.
        - Tell me about communicating with your team within Cerner.

7. **Role changes related to Cerner**
    - Have anyone's duties or roles changed as a result of how things are set up in Cerner?
    - Tell me what it's been like to work with others during the transition.
        - *Probe:* Your direct team members, others outside your direct team

8. **Support**
    - When you need help with something in Cerner, who do you ask?
    - AS NEEDED: have you used?:
        - the helpdesk or helpline
        - superusers
        - in-person support from Cerner
        - any online support available
        - your colleagues do to help each other with Cerner
        - requesting changes to Cerner
    - Did your team or clinic [other organizational unit?] do anything as a group to help you and your colleagues use Cerner?

9. **Conclusion**
    - Is there anything else you want us to know?

10 MONTHS POST-IMPLEMENTATION

Grounded probes/prompts: *If responses are limited or require clarification, probes may be used to elicit more detailed responses. Probes should use words or phrases presented by the participant using one of the following formats:*

What do you mean by ____________?
Tell me more about___________.
Give me an example of ____________.
Walk me through ___________.
Tell me about a time when ____________.
Who ____________?
Where ____________?
What, if anything, was helpful about ________________?
What, if anything, was not helpful about ________________?
What, if anything, made ________________ difficult?
What, if anything, made ________________ easier?
What was the impact of ____________?
Tell me about the _______ training session you participated in.
What's happening with _____ now?

Our last interview was a couple months after go-live, in [*month of prior interview*]. I'd like to take this chance to hear about what has changed since then.

1. **Tell me how the implementation of Cerner at your facility has been going since our last interview**
    a. **PROBE** on *previous key issues* raised in post-implementation interview
    b. **PROBE** on *changes* since the last interview

2. **Since our last interview, how has the Cerner transition impacted Veterans?**

   AS NEEDED:
   What about Veteran care?
   What about Veteran experience?
   What's happening with the patient portal?

3. **Patient Safety**
   - Tell me about patient safety since we last spoke.
   - What's the process for reporting a patient safety issue?
     - Specific events
     - General conditions

   AS NEEDED:
   - Since we last spoke, have there been patient safety issues related to the EHR transition?

4. **Information/Communication**
   - Tell me about communication regarding Cerner since we last spoke.
     AS NEEDED:
     - Tell me about any information you have received from
       - Local leaders [from chief or supervisor]
       - VA leadership
       - Cerner
       - VISN and VAMC-level leadership

5. **Training and education**
   - Since we last spoke, have you found anything that has helped you with Cerner?
   - Have you received any additional training since we last spoke? Please tell me about it.
     a. [If no]: Would you want to receive more training in using Cerner? Why or why not?

6. **Resources and capacity**
   - Tell me about clinic capacity and access for veterans since we last spoke.
     AS NEEDED:
     a. Tell me about the Clinical Resource Hub [Boise Hub]
     b. Tell me about the call center

7. **Using Cerner**
   - Since we last spoke, what has it been like for you to use Cerner to do your work?
     AS NEEDED:
     - Have you made any changes to tailor Cerner to your needs?

   - We've heard that Cerner has different functions for different roles, has that affected you at all?
     AS NEEDED:
     - Is there anything you can no longer do because of these role-based changes?
     - Has this change impacted how you work with colleagues?

   - Tell me about documentation in Cerner.
     AS NEEDED:
     - Do you document outside of your regular work hours?

Interoperability:
- Have you communicated with other VA sites through Cerner?
- Have you communicated with non-VA systems through Cerner?
- Tell me about communicating with your team within Cerner?

8. **Role changes related to Cerner**
   - Have anyone's duties or roles changed as a result of how things are set up in Cerner?
   - Do you know if anyone you worked with left VA because of the EHR transition?

9. **Support**
   - Recently, when you need help with something in Cerner, who do you ask?

10. **Time Permitting**
    - Given what you know now, what do you wish you had known or done when Spokane went live with Cerner?
    - If you were in charge of the VA, and could change anything related to the EHR transition, what would you do?
    - As you likely know, after the go-live in Spokane, Congress conducted a strategic review of the rollout, and as a result the VA has talked about making changes to the rollout process. Has any of this affected you or your facility?

11. **Conclusion**
    - Is there anything else you want us to know?
